# Bibliometric and visual analysis of microglia-related neuropathic pain from 2000 to 2021

**DOI:** 10.3389/fnmol.2023.1142852

**Published:** 2023-05-18

**Authors:** Shun-Bai Zhang, Guang-Hai Zhao, Tian-Run Lv, Chao-Yang Gong, Yong-Qiang Shi, Wei Nan, Hai-Hong Zhang

**Affiliations:** ^1^Lanzhou University Second Hospital, Lanzhou, China; ^2^Orthopaedics Key Laboratory of Gansu Province, Lanzhou, China

**Keywords:** microglia, neuropathic pain, bibliometric analysis, CiteSpace, VOSviewer, visual analysis

## Abstract

**Background:**

Microglia has gradually gained researchers’ attention in the past few decades and has shown its promising prospect in treating neuropathic pain. Our study was performed to comprehensively evaluate microglia-related neuropathic pain via a bibliometric approach.

**Methods:**

We retrospectively reviewed publications focusing on microglia-related neuropathic pain from 2000 to 2021 in WoSCC. VOS viewer software and CiteSpace software were used for statistical analyses.

**Results:**

A total of 2,609 articles were finally included. A steady increase in the number of relevant publications was observed in the past two decades. China is the most productive country, while the United States shares the most-cited and highest H-index country. The University of London, Kyushu University, and the University of California are the top 3 institutions with the highest number of publications. *Molecular pain* and *Pain* are the most productive and co-cited journals, respectively. Inoue K (Kyushu University) is the most-contributed researcher and Ji RR (Duke University) ranks 1st in both average citations per article and *H*-index. Keywords analyses revealed that pro-inflammatory cytokines shared the highest burst strength. Sex differences, neuroinflammation, and oxidative stress are the emerging keywords in recent years.

**Conclusion:**

In the field of microglia-related neuropathic pain, China is the largest producer and the United States is the most influential country. The signaling communication between microglia and neurons has continued to be vital in this field. Sexual dimorphism, neuroinflammation, and stem-cell therapies might be emerging trends that should be closely monitored.

## Introduction

Neuropathic pain is a more common but rather severe and intractable disease than any other chronic pain disease (Torrance et al., 2006), which often presented as spontaneous but persistent pain, nociceptive hyperalgesia, and allodynia (Zhao et al., 2017). The widely accepted definition of neuropathic pain is caused by an injury or disease of the somatosensory system (Jensen et al., 2011). Unlike nociceptive pain which protects against noxious stimuli, neuropathic pain is a maladaptive response to injury of the nervous system (Costigan et al., 2009). Common causes of this disorder include postherpetic neuralgia, trigeminal neuralgia, analgesic radiculopathy, diabetic neuropathy, autoimmune diseases, cancer, and neurological damage from chemotherapy or trauma (Freeman, 2009). Even more problematic is the persistence of neuropathic pain. Numerous animal studies based on peripheral nerve injury have shown that neuropathic pain results from neural plasticity. In the peripheral nervous system (PNS) and central nervous system (CNS), it is manifested as peripheral sensitization and central sensitization, respectively (Ji et al., 2003; Basbaum et al., 2009). The persistence of neuropathic pain has continued to be an intractable and debated issue, which lacks effective therapeutic modalities (Costigan et al., 2009). Analgesic modalities such as acetaminophen, NSAIDs, or weak opioids are not effective in patients with neuropathic pain (Colloca et al., 2017). The traditional approach to managing patients with neuropathic pain remains through conservative medication, with first-line management including tricyclic antidepressants, pregabalin, and gabapentin (Finnerup et al., 2015; Colloca et al., 2017; Moisset et al., 2020). However, these pharmacological treatments have been proven to be effective in less than half of patients with neuropathic pain and various adverse effects have been observed (Finnerup et al., 2015; Moisset et al., 2020), so there is an urgent need to develop new therapeutic options that are safe and provide long-term relief (Rasche et al., 2006; Morgalla et al., 2017). Basic studies and clinical trials may find drugs that target new mechanisms of action and hold promise for alleviating neuropathic pain through new therapeutic targets (Hone et al., 2018). Among these, new strategies for modulating neuron–glia interactions in neuropathic pain conditions hold considerable promise (Ahmad et al., 2021; Ruiz-Cantero et al., 2021).

For a long time, research on chronic pain has generally been centered on neurons (Grace et al., 2021). However, accumulating evidence has suggested that microglia play a key role in the development and maintenance of neuropathic pain by interacting with neurons (DeLeo and Yezierski, 2001). Microglia are macrophage-like cells in the central nervous system (Du et al., 2017). Injured tissues or neurons release various mediators that lead to microglia activation, causing morphological changes and the release of pro-inflammatory and pro-injurious mediators (e.g., pro-inflammatory cytokines and chemokines) involved in the development and maintenance of neuropathic pain (Austin and Moalem-Taylor, 2010). These mediators increase excitatory currents or decrease inhibitory currents by activating key signaling pathways (e.g., MAP kinase pathways; Detloff et al., 2008), ultimately producing neuropathic pain. Although the specific mechanisms by which microglia produce neuropathic pain have not been fully elucidated, the available evidence suggests that targeting microglia for neuropathic pain remains promising, including targeting the MAPK signaling pathway [ERK (Inoue, 2006), p38 (Ji and Suter, 2007), JNK (Ji et al., 2009)], antagonizing upstream activators of microglia [e.g., P2X4 (Inoue, 2006) and MMP-9/2 (Li et al., 2016)], targeting downstream mediators released by microglia [e.g., TNF-α, IL-1β, IL-6 (Yang et al., 2020) or BDNF (Coull et al., 2005)] and secreting anti-inflammatory (Tao et al., 2016) and analgesic mediators (Huang et al., 2017) via microglia.

Bibliometric research uses quantitative methods such as mathematics and statistics to process the characteristics of literature, which can, to a certain extent, describe, evaluate and predict the current research status and development trend of a specific subject and reflect the current research hotspots and frontiers (Wu et al., 2022). Thanks to advances in visualization technology, we used bibliometric software packages [CiteSpace (Chen, 2006) and VOSviewer (van Eck and Waltman, 2010)] to analyze and visualize potential trends in microglia-related neuropathic pain. We searched and collected relevant literature from the Web of Science core collection (WoSCC). A comprehensive analysis of this literature’s annual output, authors, countries/regions, affiliations, journal-related information, keywords, and references was also conducted (Chen and Wang, 2020). The contributions of academic groups and prominent researchers were objectively assessed to identify a fundamental overview of the field. Secondly, we identified the body of knowledge on the research topic by reviewing and analyzing the co-cited references. Finally, keyword co-occurrence and clustering analyses were used to detect hotspots and their evolution from 2000 to 2021. And CiteSpace’s Burst Detection function is used to identify emerging topics that may evolve into research hotspots in the future (Yang et al., 2022).

Reading publications on microglia in neuropathic pain, including reviews, basic research, and clinical trials, allows us to keep abreast of the latest advances and the most outstanding contributions to the field. However, the hotspots and frontiers in this field are constantly updated, so a concise summary of the relevant areas is lacking. Consequently, a comprehensive analysis and summary of the current state of research, key areas, and research perspectives on microglia-related neuropathic pain will give researchers new insight into the academic framework for grasping microglia-related neuropathic pain and help them to formulate future scientific work.

## Method

### Data acquisition and cleaning

**Figure 1:**
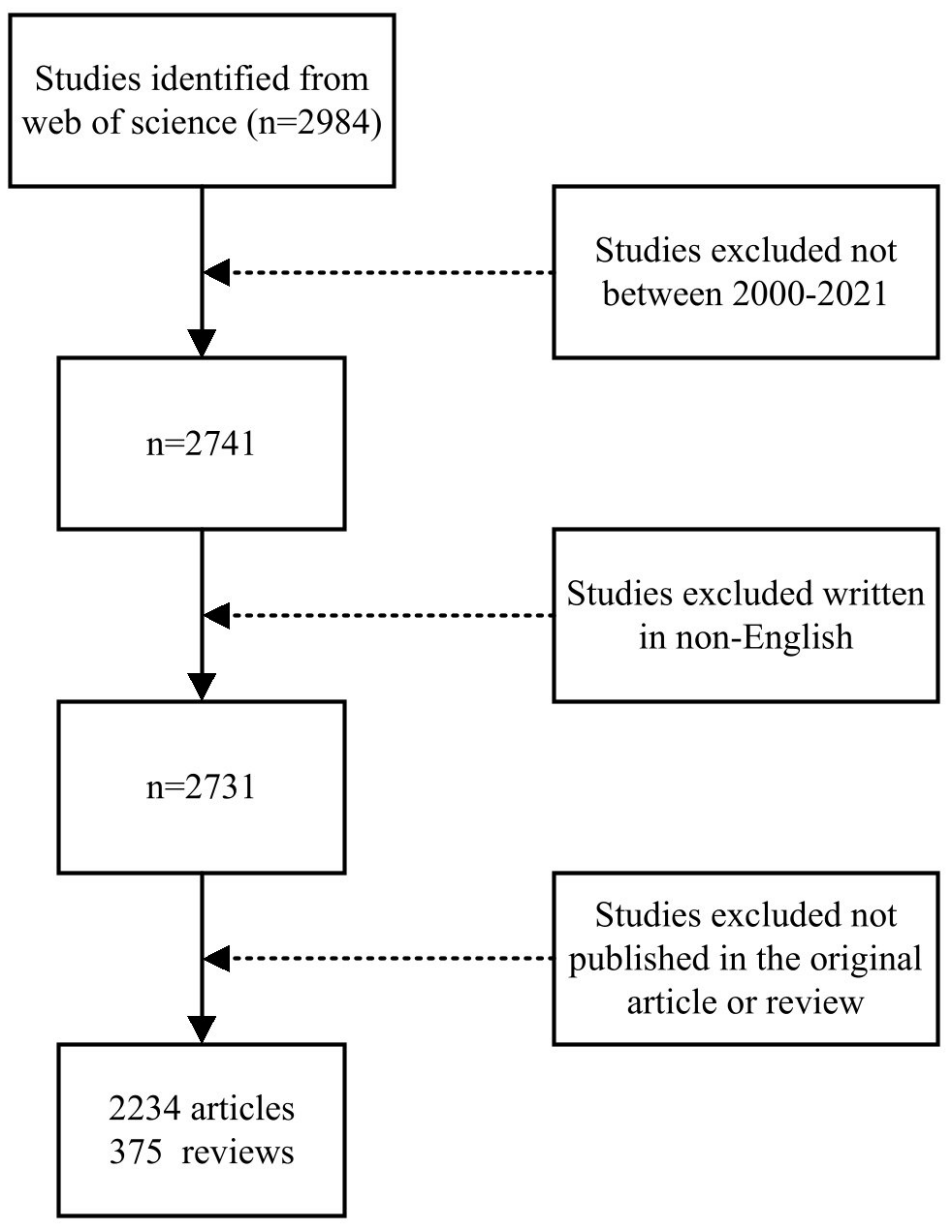
Flowchart describing the literature selection process.

The Web of Science (WoS) core database from Clarivate Analytics was used for the bibliometric analysis. To avoid bias resulting from daily database updates, all articles related to microglia in neuropathic pain from 1 January 2000 to 31 December 2021 were retrieved and downloaded from the WoS Core Collection (WoSCC) database on 10 November 2022. Using the following search strategy: TS = (Microglia*) AND TS = (“Neuropathic Pain” OR “Trigeminal Neuralgia” OR “Postherpetic Neuralgia” OR “Pain After Peripheral Nerve Injury” OR “Painful Polyneuropathy” OR “Painful Radiculopathy” OR “Painful Diabetic Neuropathy” OR “Diabetic Painful Neuropathy” OR “Chemotherapy Induced Peripheral Neuropathy” OR “Chronic Inflammatory Demyelinating Polyneuropathy” OR “Neuropathic Cancer Pain” OR “Spinal Cord Injury Pain” OR “Central PostStroke Pain”). Only research and review articles written in English were selected and other document types, such as letters, briefings, and meeting abstracts, were excluded (Figure 1). Two researchers (Zhang Shun-Bai and Zhao Guang-Hai) collected the main data separately. Any disagreements were discussed and negotiated. The search results were chosen as “Full Record and Cited References” and exported as a “Plain Text file” with download_*.txt format for storage. We create a VOSviewer thesaurus file to perform synonym merging (e.g., author name, organization name, country name), correct spelling differences, and remove meaningless words before importing the data into VOSviewer (version 1.6.18) for analysis. The cleaned data were then imported into VOSviewer for bibliometric analysis.

### Bibliometric analysis and visualization maps

This study was based on a bibliometric analysis of the number of publications and citations, *H*-index, year of publication, country/region, institution, journal, author, citations, references, and keywords in the literature related to microglia in neuropathic pain. In general, the total number of publications (NP) and the total number of citations (NC) are widely used as the two main perspectives to reflect the level of productivity and impact (Wang et al., 2021). H-index is increasingly used as a useful indicator to assess researchers’ scholarly contribution and predict future scientific achievement (Eagly and Miller, 2016). Currently, H-index is used to evaluate individual scholarly achievement and is now extended to evaluate the scholarship of a country or region, an institution, or a journal (Jones et al., 2011). In addition, the WOS Website’s online “Citation Report” function is used to obtain the latest journal impact factors (IF), which are used to measure the quality and impact of the target journal (Chen, 2006).

**Figure 2:**
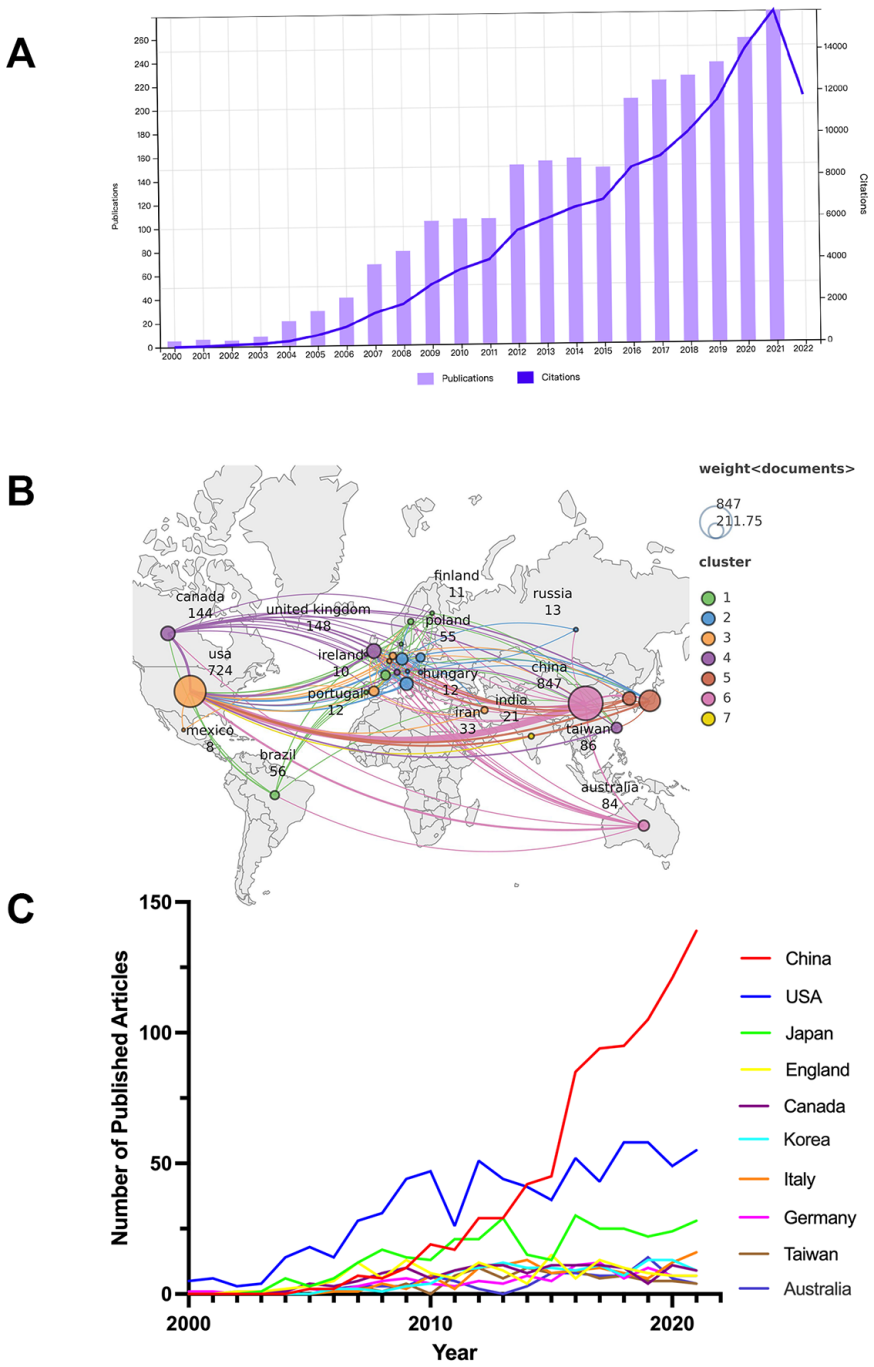
**(A)** Annual productions and citations in studies of microglia-associated neuropathic pain from 2000 to 2021. **(B)** Global distribution of national publications and intercountry cooperation related to microglia-associated neuropathic pain from 2000 to 2021. **(C)** The country’s annual trend publications related to microglia-associated neuropathic pain from 2000 to 2021.

Microsoft Excel 2019 software and GraphPad Prism (version 8.0.2) were used to collate and plot data for the annual publications. We combined Scimago Graphica (version 1.0.23), a visualization tool for exploring and communicating data (Liu et al., 2022), with VOSviewer (version 1.6.18) to map the global distribution of national publications and inter-country collaboration (Figure 2B). In addition, VOSviewer and CiteSpace (version 6.1 R4) were used for knowledge mapping in this article, each of which has its characteristics and can complement the other. Thanks to the development of the tools mentioned above, it is possible to help researchers create a knowledge structure, to understand the current state of research from a macro perspective, and to identify hotspots in target research fields. The most common types of research methods include cluster, co-occurrence, co-citation, and burst analysis. Cluster analysis is a statistical method of categorizing data according to their degree of similarity and aims to reveal the specific distribution of study content on a given topic (Ai et al., 2022). Briefly, co-occurrence analysis refers to two words frequently occurring in the same article and which may be more closely related than others. Researchers can use co-occurrence analysis to identify trends and hot spots in a subject. Small (1973) initially proposed a method of co-citation analysis in 1973 to analyze the relationships and structure of academic fields. Unlike citation analysis, which evaluates the quality of a topic by the number of citations, co-citation analysis helps scholars quickly discover the structure and characteristics of a target field of research by screening out a representative selection of documents for analysis and then using citation network analysis to display them into various clusters (Liao et al., 2018). Currently, the co-citation analysis has been widely used to reveal the relationships between authors, articles, and journals (Liao et al., 2018). Burst analysis is an algorithm developed by Kleinberg to understand the rapid growth in the popularity of references or keywords over a defined period of time (Kleinberg, 2003). Citation Burst analysis enables researchers to understand how fast-changing literature transforms the knowledge landscape of a scientific field and reveals the cutting-edge research junctions that are active today (Chen, 2006).

VOSviewer, developed by Eck and Waltman, uses a probabilistic-based approach to data normalization and provides a variety of visualization views in the areas of co-authorship, co-citation, keywords, etc., including Network Visualization, Overlay Visualization, and Density Visualization, which provides easy mapping and beautiful graphics for large bibliometric maps, as well as powerful features for co-occurrence analysis, co-citation analysis, and bibliographic coupling analysis (van Eck and Waltman, 2010). VOSviewer software was used to systematically analyze and visualize the distribution and collaboration of countries/regions, institutions, journals, authors, and co-occurrence of keyword clusters. In VOSviewer maps, the node’s size indicates the total number of co-citations, co-authorship, co-occurrence, or bibliographic coupling for that item. Larger nodes indicate more contributions for that item; the largest nodes are highlighted in red. A line between two nodes indicates that both items have been cited in the same document, and a shorter line indicates a closer relationship between the two items. The thickness of the line generally indicates the degree of co-occurrence, co-citations, and co-authorship (Gu et al., 2017). CiteSpace (5.6.R3) software, a bibliometric visualization tool developed by Chen, is widely used to analyze metrics such as countries, institutions, authors, journals, keywords, etc. (Chen et al., 2012) and is particularly known for its Burst Detection feature. In this study, CiteSpace was used to extract Citation Bursts for references and keywords to help identify research hotspots, current research status, and trends in the field (Chen, 2004). In the citation bursts for references and keywords, the blue line indicates the period in the graph, while the red line represents the period in which the reference burst occurred.

## Result

### Annual trend of publications

The total number of annual publications (NP) over a period of time provides an objective indicator of the overall trends in a field. A total of 2,609 publications on microglia in neuropathic pain were published in the WoSCC from 1 January 2000 to 31 December 2021, including 2,234 papers (85.63%) and 375 reviews (14.37%). The literature covered 66 countries or regions and 1754 institutions. Figure 2A shows the NP on microglia in neuropathic pain. From 2000 to 2003, the number of articles published on related research was low. From 2004 to 2009, the NP increased rapidly as more scholars focused on the research mechanisms in this field, with the NP reaching 104 in 2009. What is striking is that in 2012 and 2016, there was a “boom” in article production. But with the end of these two “booms” came a slowdown in article production. The number of articles published yearly has increased from 106 in 2010 to 279 in 2021, and the growth rate has remained relatively stable. The 2,609 publications have been cited 120,112 times to date, excluding self-cited articles numbered 91,295, with an average citation frequency of 46.04 per article. Annual citations from 2000 to 2021 show a consistently increasing trend, as shown in Figure 2A. The *H*-index is 151, which indicates a considerable number of highly cited articles in this research field.

### Countries

A total of 66 countries/regions were covered in the 2,609 articles, of which 27 countries/regions published more than 10 articles (Figure 2B). The top 10 most influential countries/regions are listed in Table 1, along with their total number of publications (NP), total citations (NC), average citation frequency (AC), and H-index. The top 10 countries/regions published 87% (2,279/2609) of the publications. China was the leading country in terms of total publications (32.5%, 847/2609), followed by the United States (27.9%, 727/2609) and Japan (12.5%, 326/2609). The top three countries with the highest total number of citations were the United States (52,361), China (20,243), and Japan (15,970). The top three countries with the highest average citation frequency were the United Kingdom (84.76), Canada (82.07), and Australia (71.37). The top three countries in the *H*-index were the United States (Peng et al., 2021), the United Kingdom (Clark et al., 2007), and Japan (Milligan et al., 2004), with China (Zhuang et al., 2007) following closely behind.

**Table 1:** Top 10 most productive countries in microglia-related neuropathic pain from 2000 to 2021.

Rank	Country	NP	NC	AC	*H*-index	Number of cooperating countries
1	China	847	20,243	23.9	65	22
2	United States	727	52,361	70.02	117	30
3	Japan	325	15,970	49.14	66	13
4	England	148	12,531	84.67	67	25
5	Canada	144	11,818	82.07	61	20
6	Korea	125	3,879	31.03	36	7
7	Italy	120	5,798	48.32	45	17
8	Germany	100	5,399	53.99	40	18
9	Taiwan	86	2,683	31.2	28	7
10	Australia	84	5,995	71.37	37	14

Annual publication trends for the top 10 countries in terms of output from 2000 to 2021 are shown in Figure 2C. Research on microglia-related neuropathic pain in the United States is central to global research, and research in China has become more active in the last decade. China initially lagged behind the US in the annual output of publications, but publications in this area have grown rapidly since 2012, overtaking the US in 2014, and will continue to grow rapidly through 2021. In addition, the US and Japan show a “fluctuating upward trend” in the annual output of publications.

Figure 2B also illustrates the mapping of national collaborations. It can be seen that China has established international cooperation with several countries/regions, with the US being the most collaborative, followed by Japan, Germany, the United Kingdom, Canada, Australia, and South Korea. The countries/regions with the highest number of publications (China, United States, Japan, United Kingdom, and Canada) also cooperate more closely.

### Institutions

Over 1755 institutions contributed to this field, with 110 publishing more than 10 papers. Table 2 summarizes the top 10 most impactful institutions, from China (3/10), the United States (3/10), the United Kingdom (2/10), Japan (1/10) Canada (1/10). The University of London (Bennett et al., 2016), Kyushu University (Soliman et al., 2021), University of California (Clark et al., 2009), and Shanghai Jiao Tong University (Clark et al., 2009) are the top 4 institutions in terms of the number of papers published, which is representative of the research capacity of the scientific institution in its field of study. The top 3 institutions in average citation frequency were Harvard University (AC = 161.23), the University of Colorado System (AC = 121.89), and the University of Toronto (AC = 111.64). And the top 3 research institutions in terms of H-index were the University of London (Tsuda et al., 2004), Kyushu University (Chen, 2004), and Harvard University (Small, 1973).

**Table 2:** Top 10 productive institutions in microglia-related neuropathic pain from 2000 to 2021.

Rank	Institution	Country	NP	NC	AC	*H*-index
1	University of London	England	85	8,220	96.71	51
2	Kyushu University	Japan	82	7,771	94.77	45
3	University of California System	USA	62	3,538	57.06	32
4	Shanghai Jiao Tong University	China	62	1,239	19.98	22
5	Harvard University	England	60	9,674	161.23	40
6	University of Toronto	Canada	58	6,475	111.64	38
7	Fudan University	China	58	2,839	48.95	28
8	University of Colorado System	United States	55	6,704	121.89	35
9	University of Texas System	United States	55	3,002	54.58	29
10	Sun Yat-sen University	China	53	2,476	46.72	24

**Figure 3:**
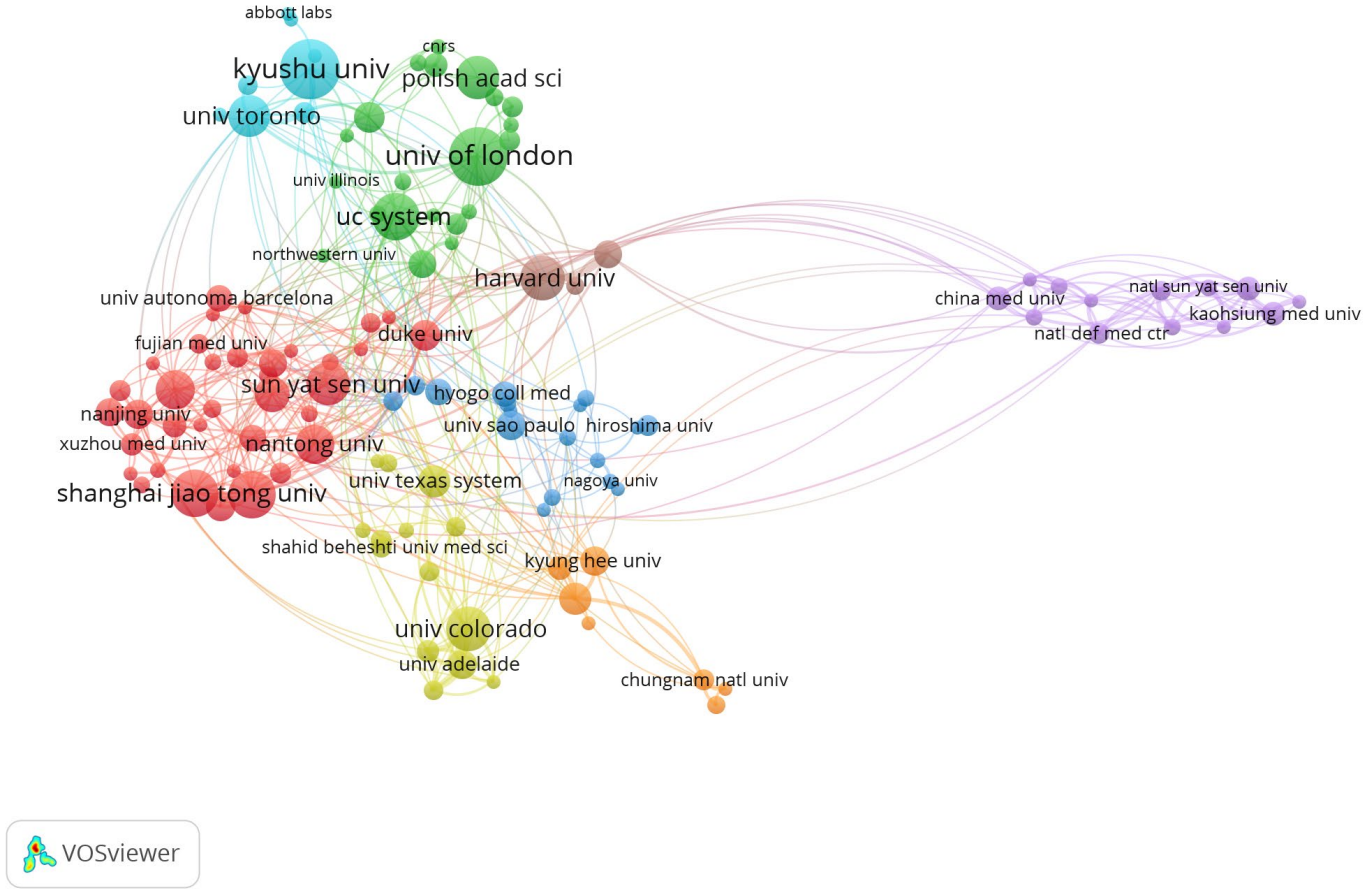
Analysis of the clustering of publications from different institutions.

Cross-institutional collaboration can facilitate in-depth research in the field. Analysis of the institutional collaboration network mapping shows that the 119 institutions with more than 10 occurrences (threshold >10 papers) are distinguished into 8 clusters by different colors (Figure 3). According to the clustering analysis, the University of California System (Inoue, 2006), Harvard University (Detloff et al., 2008), the University of Toronto (DeLeo and Yezierski, 2001), the University of Texas System (DeLeo and Yezierski, 2001), the University of London (Grace et al., 2021), Fudan University (Ruiz-Cantero et al., 2021), Shanghai Jiao Tong University (Hone et al., 2018) and Kyushu University (Rasche et al., 2006) have collaborated and communicated extensively on the mechanisms of microglia in neuropathic pain.

It is evident that the above institutions not only publish a large number of articles but also appear to have more widespread collaborations with others. US institutions often conduct their research with a transnational approach. Strong collaborations exist between the University of Toronto, the University of London, Shanghai Jiao Tong University, and Kyushu University. In contrast, cross-institutional collaboration in other countries is mainly through intra-national work. In China, for example, there is close collaboration between the Fourth Military Medical University, Capital Medical University, Wenzhou Medical University, and Fujian Medical University (Figure 3).

### Funding source

Adequate financial support plays a vital role in the development and advancement of science. Table 3 summarizes the top 10 funding agencies and sponsors in this field. The National Natural Science Foundation of China (NSFC) is the major funding agency in China. Half of the top 10 funding agencies, including the US Department of Health and Human Services (HHS), the National Institutes of Health (NIH), the NIH National Institute of Neurological Disorders Stroke (NINDS), the National Institute on Drug Abuse (NIDA) and the National Institute of Dental Craniofacial Research (NIDCR), are from the US. Meanwhile, Japanese research funding agencies, including the Grants in Aid for Scientific Research (KAKENHI) and Science and the Japan Society for the Promotion of Science (JSPS), reflect the strong research capacity of Japanese research institutions in this field. It is worth noting that the European Commission provides major funding support for European countries (including the United Kingdom, Italy, Germany, etc.). With sufficient funding, the US has maintained a leading position in microglia and neuropathic pain research.

**Table 3:** The top 10 funding sources with the most publications.

Rank	Funding source	Country	NP	NC	AC	*H*-index
1	National Natural Science Foundation of China (NSFC)	China	526	12,761	24.26	54
2	United States Department of Health and Human Services (HHS)	USA	471	40,838	86.7	108
3	National Institutes of Health (NIH)	USA	470	40,861	86.84	108
4	European Commission	EU	267	22,400	83.9	85
5	NIH National Institute of Neurological Disorders Stroke (NINDS)	USA	232	24,740	106.64	85
6	Ministry of Education Culture Sports Science and Technology Japan (MEXT)	Japan	186	6,546	35.19	46
7	NIH National Institute on Drug Abuse (NIDA)	USA	148	13,652	92.24	62
8	Japan Society for The Promotion of Science (JSPS)	Japan	135	4,079	30.21	37
9	NIH National Institute of Dental Craniofacial Research (NIDCR)	USA	99	14,941	150.92	62
10	Grants in Aid for Scientific Research (KAKENHI)	Japan	94	2,488	26.47	27

### Journals and co-cited journals

A total of 540 journals published articles on microglia and neuropathic pain. Of these, 111 journals contributed at least five papers. The top 10 most influential journals are listed in Table 4. The research interests of these journals focus on pain, inflammation, and immunity. Seven publishers are located in the United States; the remainder is from the Netherlands, United Kingdom, and Ireland. *Molecular pain* was the most prolific journal (109 articles, 4.178%), followed by *Pain* (107 articles, 4.101%) and the *Journal of Neuroscience* (81 articles, 3.105%). *Brain Behavior and Immunity* had the highest impact factor (19.227), followed by the *Journal of Neuroinflammation* (9.587) and *Pain* (7.926). *Journal of Neuroscience* had the highest total citations (10,549) and average citation frequency per article (130.23). Furthermore, five of the top 10 journals are in the Q1 JCR division, two are in the Q2 JCR division and the remaining three are from the Q3 JCR division.

**Table 4:** The top 10 productive journals of microglia in neuropathic pain research.

Rank	Journal	Country	NP	NC	AC	*H*-index	IF (2021)	Quartile in category
1	Molecular pain	United States	109	4,078	37.41	38	3.370	Q3
2	Pain	United States	107	8,189	76.53	50	7.926	Q1
3	Journal of Neuroscience	United States	81	10,549	130.23	57	6.709	Q1
4	Brain Behavior and Immunity	Netherlands	77	3,916	50.86	34	19.227	Q1
5	Journal of Neuroinflammation	England	76	2,505	32.96	33	9.587	Q1
6	Neuroscience	United States	76	3,623	47.67	35	3.708	Q3
7	Neuroscience Letters	Ireland	53	1,224	23.09	22	3.197	Q3
8	Experimental Neurology	United States	50	3,015	60.3	33	5.62	Q2
9	Journal of Pain	United States	43	1941	45.14	27	5.383	Q1
10	Plos One	United States	43	1,516	35.26	26	3.752	Q2

VOSviewer found that 121 of the 5,916 co-cited journals reached the threshold (minimum number of citations >200). Of these, 32 journals had more than 1,000 co-citations and 14 had more than 2,000. According to Table 5, *Pain* was the most co-cited journal (10,958), followed by the *Journal of Neuroscience* (10,337), *Glia* (3,625), *Neuroscience* (3,573), and *Proceedings of the National Academy of Sciences of the United States of America* (3,223), all five journals with publishers from the US. *Nature* has the highest impact factor (69.504) among the top 10 co-cited journals. Additionally, five of the top 10 co-cited journals are in the Q1 JCR division, two in the Q2 JCR division, and the remaining three in the Q3 JCR division.

**Table 5:** The top 10 co-cited journals of microglia in neuropathic pain research.

Rank	Co-cited journal	Country	Citation	IF (2021)	Quartile in category
1	Pain	United States	10,958	7.926	Q1
2	Journal of Neuroscience	United States	10,337	6.709	Q1
3	Glia	United States	3,625	8.073	Q1
4	Neuroscience	United States	3,573	3.708	Q3
5	Proceedings of the National Academy of Sciences of the United States of America	United States	3,233	12.779	Q1
6	Brain Research	Netherlands	3,112	3.61	Q3
7	Nature	England	2,840	69.504	Q1
8	Experimental Neurology	United States	2,775	5.65	Q2
9	Molecular pain	United States	2,636	3.370	Q3
10	Journal of Neurochemistry	England	2,560	5.546	Q2

### Authors and co-cited authors

Over 8,964 authors have published in this field of research. Of these, 81 authors have contributed over 10 papers to the field. Table 6 summarizes the top 10 academics regarding the number of publications. Inoue K from Kyushu University is the most prolific author (NP = 61, NC = 6,223, H-index = 42), and Ji RR from Duke University has the highest average citation frequency (AC = 206.44) and the highest H-index (Gu et al., 2017). It is worth noting that the top two authors in terms of publications both belong to Kyushu University and have collaborated many times.

**Table 6:** The top 10 contributed authors in microglia-related neuropathic pain from 2000 to 2021.

Rank	Author	Affiliations	Country	NP	NC	AC	*H*-index
1	Inoue K	Kyushu University	Japan	61	7,223	118.41	42
2	Tsuda M	Kyushu University	Japan	60	6,389	106.48	39
3	Watkins LR	University of Colorado System	United States	53	6,665	125.75	35
4	Ji RR	Duke University	United States	50	10,322	206.44	43
5	Mika J	Polish Academy of Sciences	Poland	48	2,371	49.4	27
6	Maier SF	University of Colorado System	United States	41	5,095	124.27	31
7	Zhang Y	University of Colorado System	United States	39	1,278	32.77	18
8	Malcangio M	University of London	England	35	3,746	107.03	27
9	Makuch W	Polish Academy of Sciences	Poland	34	1,433	42.15	22
10	Rojewska E	Polish Academy of Sciences	Poland	34	1,496	44	23

**Figure 4:**
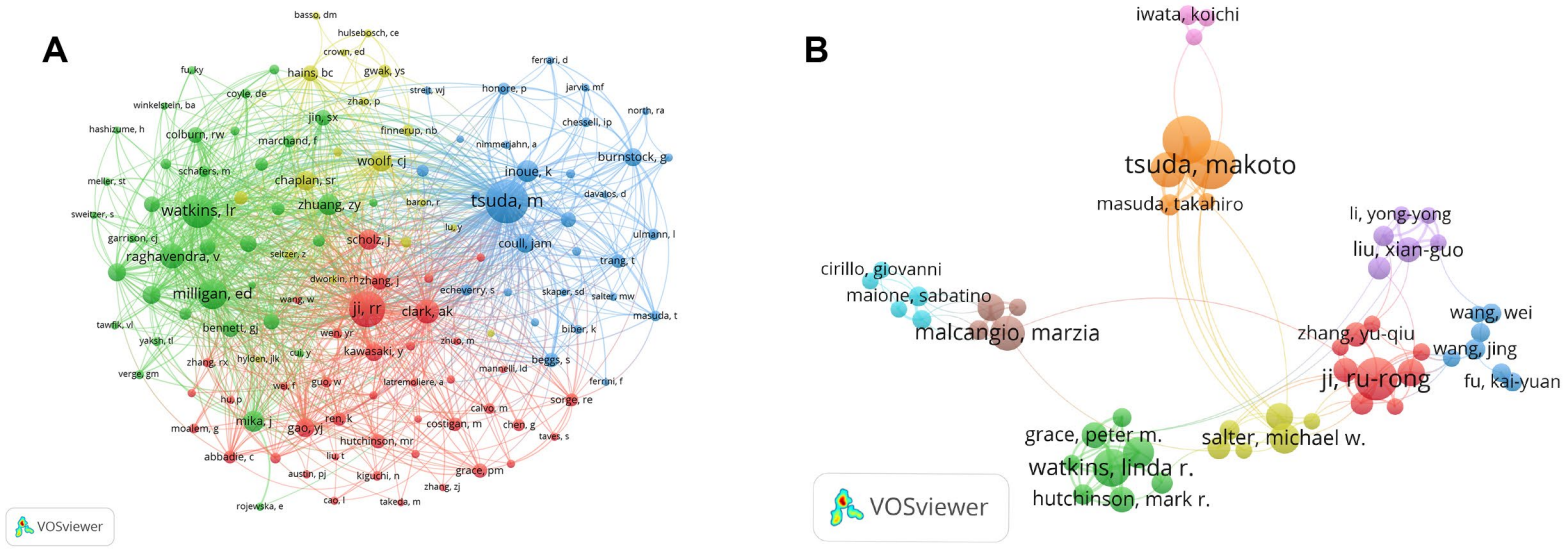
The visualization map of co-citing and co-occurring authors for microglia-associated neuropathic pain. **(A)** Visualization map of co-citing authors. **(B)** Visualization map of co-occurring authors.

Co-cited authors are two or more authors cited simultaneously in one or more papers (Xu et al., 2022). Figure 4A illustrates the network mapping of co-cited authors. The most prominent nodes are associated with the most frequently cited authors. Table 7 shows that among the 45,695 cited authors, 126 authors (grouped into 4 clusters in the map) were cited more than 100 times. In comparison, only 4 authors were cited more than 1,000 times, namely Tsuda M (1879 citations), Ji RR (1,368 citations), Watkins LR (1,207 citations) and Milligan ED (1,036 citations).

**Table 7:** The top 10 co-cited authors in microglia-related neuropathic pain from 2000–2021.

Rank	Author	Affiliations	Country	Citation	Total link strength
1	Tsuda M	Kyushu University	Japan	1879	8,604
2	Ji RR	Duke University	United States	1,368	8,452
3	Watkins LR	University of Colorado System	United States	1,207	6,736
4	Milligan ED	University of New Mexico	United States	1,036	5,489
5	Raghavendra V	Dartmouth Medical School	United States	748	4,327
6	Clark AK	University of London	England	712	4,180
7	Inoue K	Kyushu University	Japan	614	2,888
8	Zhuang ZY	Harvard University	United States	579	3,937
9	Woolf CJ	Harvard University	United States	575	3,397
10	Mika J	Polish Academy of Sciences	Poland	559	2,498

The co-occurrence of authors was mapped using VOSviewer to identify collaborative relationships between authors (Figure 4B). The map shows that in the field of microglia-related neuropathic pain, there are several collaborative sub-networks with the scholars mentioned above as the core, with varying degrees of connectivity outside the sub-networks, but with strong collaborative links between the scholars within the sub-networks. It can be seen that the Watkins LR sub-network is less collaborative with the other sub-networks, with the reduction of clinically relevant pathological pain being the main focus of this sub-network.

### Co-cited reference and reference burst

Table 8 shows the top 10 most frequently co-cited articles, with “Quantitative assessment of tactile allodynia in the rat paw” (495 citations) published in the *Journal of Neuroscience Methods* (IF = 2.987) being the most co-cited article. In addition, two of the top 10 articles were written by Tsuda M and published in different journals. Overall, four of the top 10 co-cited articles were review articles and six were basic studies. The chronological distribution in Table 8 gives a general picture of the progression of microglia in neuropathic pain research. The majority of the top co-cited articles were published between 1980 and 2018, and those with >300 co-citations were written before 2010.

**Table 8:** The top 10 co-cited references for microglia research in neuropathic pain.

Rank	Title	Journal IF (2020)	First author	Publication time	Country	Co-Citation	Quartile in category
1	Quantitative assessment of tactile allodynia in the rat paw	Journal of Neuroscience Methods (IF = 2.987)	Chaplan SR	July, 1994	United States	495	Q3
2	P2X4 receptors induced in spinal microglia gate tactile allodynia after nerve injury	Nature (IF = 69.504)	Tsuda M	August, 2003	Japan	479	Q1
3	BDNF from microglia causes the shift in neuronal anion gradient underlying neuropathic pain	Nature (IF = 69.504)	Coull Jeffrey AM	December, 2005	Canada	391	Q1
4	Inhibition of microglial activation attenuates the development but not existing hypersensitivity in a rat model of neuropathy	Journal Pharmacology Experimental Therapeutics (IF = 4.402)	Raghavendra V	August, 2003	United States	375	Q2
5	The neuropathic pain triad: neurons, immune cells and glia	Nature Neuroscience (IF = 28.771)	Scholz J	November, 2007	United States	371	Q1
6	Neuropathic pain and spinal microglia: a big problem from molecules in “small” glia	Trends Neuroscience (IF = 16.978)	Tsuda M	February, 2005	Japan	364	Q1
7	p38 mitogen-activated protein kinase is activated after a spinal nerve ligation in spinal cord microglia and dorsal root ganglion neurons and contributes to the generation of neuropathic pain	Journal Neuroscience (IF = 6.709)	Jin Shan-Xue	May, 2003	United States	362	Q1
8	Pathological and protective roles of glia in chronic pain	Nature reviews Neuroscience (IF = 38.755)	Milligan ED	January, 2009	United States	360	Q1
9	Glial activation: a driving force for pathological pain	Trends Neuroscience (IF = 16.978)	Watkins LR	August, 2001	United States	308	Q1
10	Minocycline attenuates mechanical allodynia and proinflammatory cytokine expression in rat models of pain facilitation	Pain (IF = 7.926)	Ledeboer A	May, 2005	United States	300	Q1

**Figure 5:**
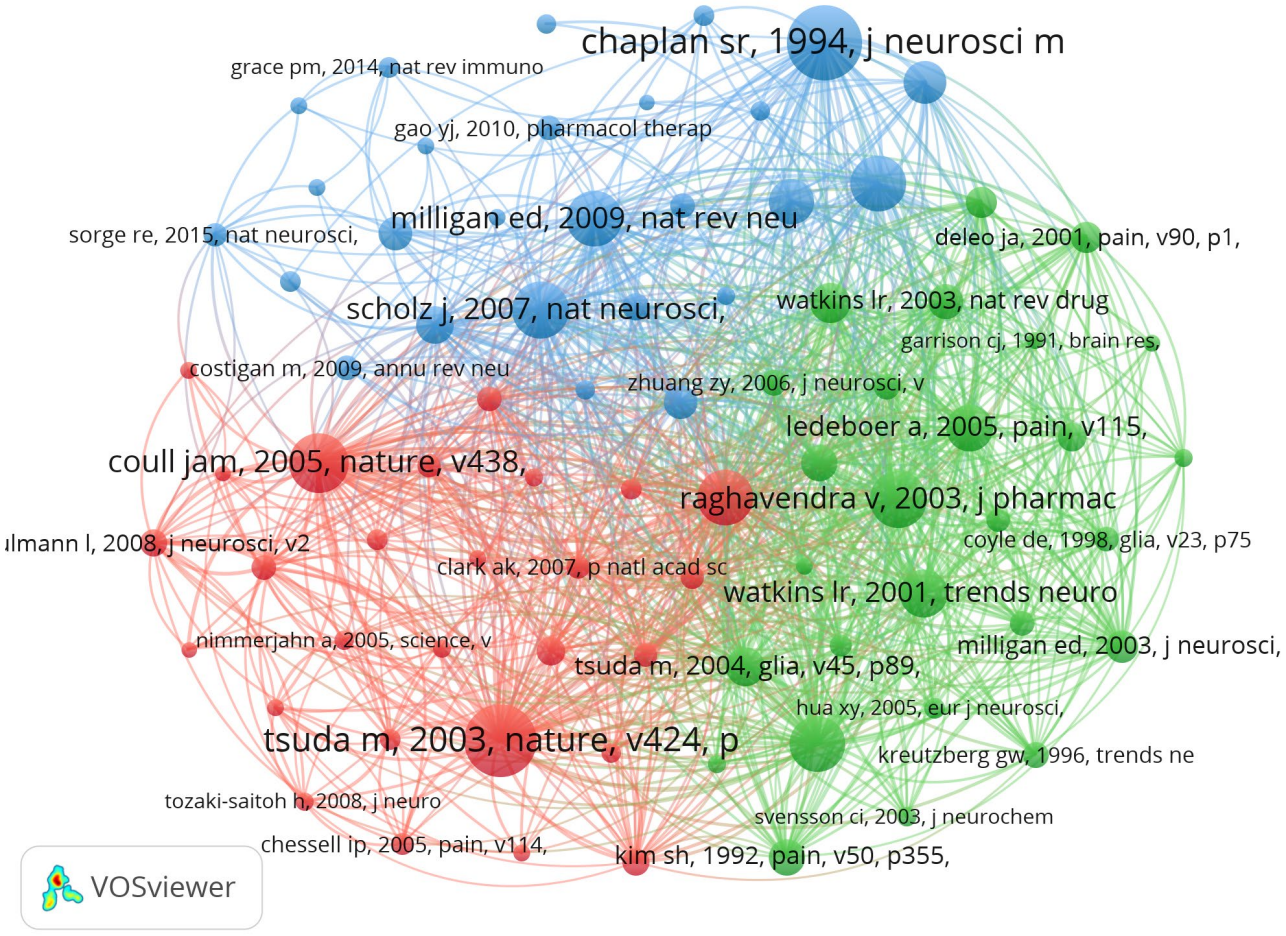
A visualization map of co-cited references on microglia-associated neuropathic pain.

A total of 72,619 references were analyzed using VOSviewer software, with a minimum total number of citations set at 100, and a total of 80 references were included. The co-citation correlations were analyzed and visualized in Figure 5. In addition, VOSviewer divided the 80 references into 3 clusters, represented by red, green and blue. The red cluster consisted of 27 papers that examined the neuroimmune role of microglia in neuropathic pain; the green cluster consisted of 27 papers that focused on the intracellular signaling mechanisms of microglia in neuropathic pain, and the blue cluster consisted of 26 papers that mainly focused on the molecular mechanisms of microglia in neuropathic pain.

**Figure 6:**
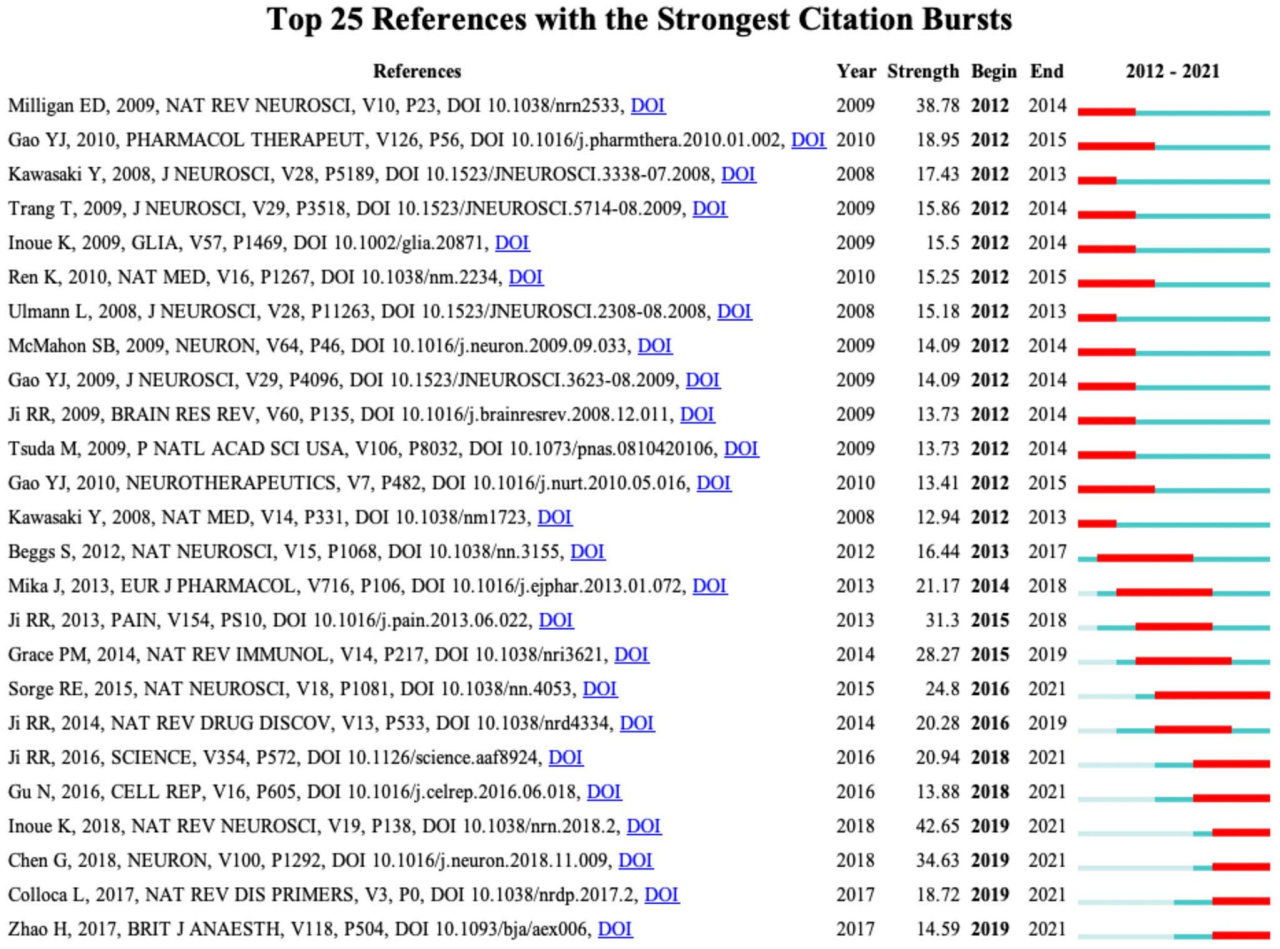
CiteSpace visualization map of the top 25 references involved in microglia-associated neuropathic pain with the strongest citation bursts.

CiteSpace performed a burst analysis of the key references and keywords in our study. The top 25 references were ranked according to the strength of their burst, as shown in Figure 6. The reference with the earliest year of burst (burst years, 2012 to 2014) was published by Milligan ED and Watkins LR in 2009 (Milligan and Watkins, 2009). The strongest burst reference was a review article entitled “Microglia in neuropathic pain: cellular and molecular mechanisms and therapeutic potential” published in 2018 (Inoue and Tsuda, 2018), with a burst strength of 42.64 and a burst year of 2019–2021. Notably, among the top 25 references, Ji RR et al. wrote four articles with strong burst values.

### Keyword co-occurrence, clusters, and burst

As an important part of the article, keywords embody the core ideas of academic research and enable us to quickly grasp the highlights and directions of the research (Li et al., 2016). By cleaning the keywords based on VOSviewer, 7,641 were retrieved from 2,609 documents. The keywords were prioritized by occurrence and link strength, as shown in Table 9, such as “microglia”(occurrence = 1,256, total link strength = 7,678), “spinal cord” (occurrence = 666, total link strength = 4,256), “activation” (occurrence = 535, total link strength = 3,908), “rat”(occurrence = 509, total link strength = 3,177), “peripheral nerve injury”(occurrence = 385, total link strength = 2,503), “mechanical allodynia”(occurrence = 377, total link strength = 2,555), “inflammation”(occurrence = 318, total link strength = 1961), “dorsal-root ganglia”(occurrence = 298, total link strength = 1862), all topped the list, suggesting that the mechanism of microglia in neuropathic pain has been a continuous hotspot in the last two decades.

**Table 9:** Top 20 keywords of microglia in neuropathic pain research.

Rank	Keyword	Occurrences	Total link strength	Rank	Keyword	Occurrences	Total link strength
1	Neuropathic pain	1884	10,456	11	Nerve injury	291	1919
2	Microglia	1,256	7,678	12	Pain	256	1,549
3	Spinal cord	666	4,250	13	Astrocytes	246	1735
4	Activation	535	3,098	14	Glial activation	238	1,677
5	rat	509	3,177	15	Hyperalgesia	237	1,598
6	Expression	478	2,860	16	Neuroinflammation	233	1,368
7	Peripheral nerve injury	385	2,503	17	Allodynia	221	1,494
8	Mechanical allodynia	377	2,555	18	Mechanisms	218	1,276
9	Inflammation	318	1961	19	Model	212	1,273
10	Dorsal-root ganglia	298	1862	20	Neurons	211	1,417

**Figure 7:**
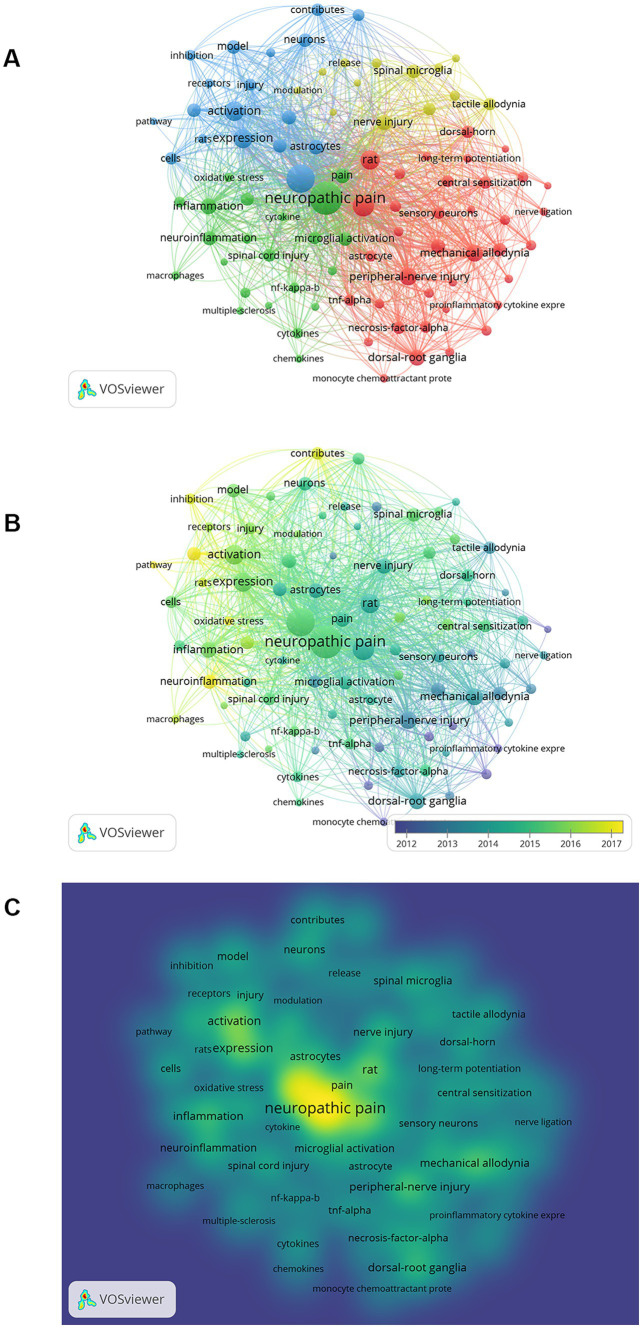
The visualization map on keywords of microglia-associated neuropathic pain. **(A)** Cluster analysis according to the occurrence of keywords. **(B)** Keyword timeline view. **(C)** Density visualization map of keywords.

A keyword co-occurrence network map was constructed, including a cluster map, a time overlay map, and a density map (Figure 7A). A total of 90 high-frequency keywords (≥50 occurrences) were detected by VOSviewer, of which 5 keywords had >500 occurrences. The nodes with the same color belong to a cluster, which shows that the research directions in this field are divided into 4 categories. The red clusters (37 items) are mainly related to cellular and molecular mechanisms of microglia in neuropathic pain, such as “central sensitization of pain,” “activation of glial cells,” “p38MAPK,” “tumor necrosis factor-α,” etc.; the green clusters (22 items) mainly related to inflammation in the CNS, e.g., “spinal cord injury,” “inflammation,” “neuroinflammation,” “activation of microglia,” “CNS,” “Alzheimer’s disease,” “cytokines,” etc.; the blue clusters (18 items) mainly related to pathophysiological mechanisms of neuropathic pain, such as “hypersensitivity,” “hyperalgesia,” “allodynia,” “mechanisms,” etc.; the yellow clusters (13 items) focus on microglia and ATP receptors, such as “release,” “up-regulation,” “ATP,” “P2X4 receptors,” “P2X7 receptors,” “neurotrophic factors,” etc.

The evolution of keywords over time was analyzed to obtain a comprehensive picture of the frontiers and hotspots in microglia-related neuropathic pain. The average time of the appearance in the document was overlayed on the keyword co-occurrence network to obtain a time overlay map (Figure 7B). The different colored nodes represent the average time that the keywords appeared in the literature, and the evolution of the research trends over time can be observed through this color variation. Clusters with more cyan nodes indicate the hotspots in this area around 2012, and clusters with more green and yellow nodes represent the latest research hotspots.

Density visualizations are uniquely useful in helping to understand the overall structure of the map and in quickly noting the most critical areas of the map. Each node in the keyword density map is colored according to the density of the items around that node. The density size is dependent on the number of surrounding items and the weighting of those items. Blue indicates low-density areas, and red represents high-density areas. It is obvious from Figure 7C that the highlights of microglia in neuropathic pain include the study of neuropathic pain, microglia, activation, inflammation, injury, and activation of glial.

**Figure 8:**
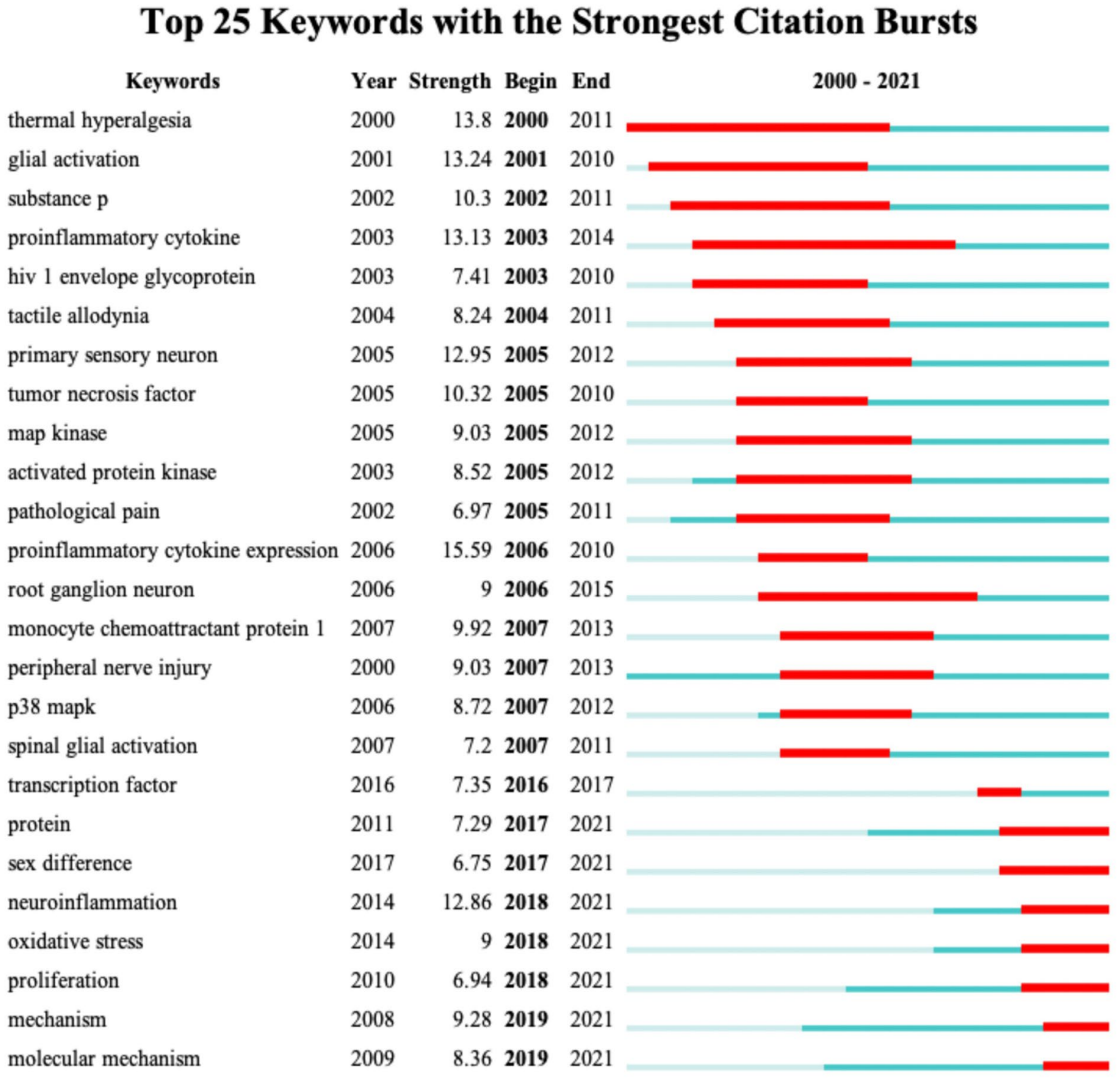
CiteSpace visualization map of the top 25 keywords involved in microglia-associated neuropathic pain with the strongest citation bursts.

Figure 8 shows the top 25 keywords with the citation burst strength. The keywords with the highest burst strength before 2010 included “thermal hyperalgesia,” “glial activation,” “substance P,” “proinflammatory cytokine,” “HIV-1 envelope glycoprotein,” “tactile allodynia,” “primary sensory neuron,” “tumor necrosis factor,” “MAP kinase,” and “peripheral nerve injury.” Focusing on the keywords that appeared after 2010, we identified emerging trends in microglia-related neuropathic pain, including “transcription factors,” “sex difference,” “neuroinflammation,” “oxidative stress,” “proliferation” and “molecular mechanism.” In addition, the keyword with the highest burst strength was “expression of pro-inflammatory cytokines”; the keyword with the longest burst duration was “thermal hyperalgesia.”

## Discussion

### General information on microglia-related neuropathic pain research

From 2000 to 2021, a total of 2,609 original research and reviews were obtained by searching the WoSCC database. In terms of annual NP, a general upward trend was observed, but after 2009 and 2012 there were 2–3 years of stagnant growth. The lack of ground-breaking research is the primary reason for the stagnation. Between 2016 and 2021, annual NP enters a period of steady growth. It is expected that the literature in this field will likely continue to grow, and the research will become more in-depth (Figure 2A).

Of the top-ranked countries, China ranks 1st in publication output (Figures 2B,C and Table 1). The United States, on the other hand, has been the driver of the highest academic contribution in this field for the past 22 years, as demonstrated by the combined performance of NP, AC, and H-index. Consistent with its economic strength, three US affiliates, five US funding agencies, and four US authors rank among the top 10 affiliates, funding sources, and authors, respectively, in studies of microglia-associated neuropathic pain (Tables 2, 3, 6). This indicates that the US has the best institutions, adequate financial support, and elite scholars, which largely account for the US being a leader in this field over the last 22 years. Similarly, Japan (AC = 49.14, *H*-index = 66) and the UK (AC = 84.67, *H*-index = 67), and Canada (AC = 82.07, *H*-index = 61) consistently maintain world-leading levels of academic productivity, suggesting that, in addition to adequate research funding, these countries keep a sustained focus and interest in the field. Compared to the countries mentioned above, China has seen a dramatic increase in NP over the past 22 years and has been No. 1 since 2014 when it overtook the US. Its relatively low AC, however, suggests that scholars from Chinese institutions may require more in-depth work to enhance the quality of their research to reinforce their international impact.

The top 10 productive institutions in microglia-related neuropathic pain were all from the top 5 countries in terms of publications (United States, China, United Kingdom, Japan, and Canada), indicating that the 5 countries play a leading role in the academic development of the field. At 9,674 total citations and 161.23 average citations, Harvard University is well ahead of the other institutions (Table 2). Much of the credit goes to the three highly cited authors (Table 7), Ji RR, Zhuang ZY, and Woolf CJ, who listed Harvard University as an affiliation of their own in many of the articles. Notably, Shanghai Jiao Tong University is the most productive university in China, ranking fourth among the Top 10 productive institutions, with the lowest average citation rate among the top 10 institutions, keeping with China’s performance on national academic influence.

Scientific research strength is built on an economic foundation. As the second-largest economy, financial support for medical research in China is continuing to increase alongside its rapidly growing economy. The National Natural Science Foundation of China (NSFC) contributed to most publications (Table 3). In addition to financial factors, the role of international academic cooperation is also crucial, with China ranking third in the number of cooperating countries. All these reasons are probably the primary contributors to the rapid and sustained growth of NP in China over the last two decades. Predictably, China’s influence on global academic productivity will be growing.

*Pain* and the *Journal of Neuroscience* are in the top three regarding both NP and co-citations, implying a significant role in microglia-related neuropathic pain (Tables 4, 5). And papers published in high IF journals such as *Nature*, *Brain Behavior and Immunity*, *Proceedings of the National Academy of Sciences of the United States of America,* and *Journal of Neuroinflammation*, this means that there will be more co-citations, potentially providing a theoretical basis for more extensive and in-depth research. It is notable, however, that among these journals, the most highly published journal, Molecular pain, has a low impact factor (3.370), as well as 40% of the highest-published journals and 30% of the highly co-cited journals with an impact factor of <4. In addition to impact factors, we should therefore include indicators other than citation-based ones to complement the appraisal of scientific accomplishment in assessing the academic value of journals (Ioannidis et al., 2014). These influential journals include a considerable amount of basic research on microglia in neuropathic pain, which means that the study of this field is still mainly focused on basic research, namely translational research from basic to clinical has not yet become a mainstream research direction.

In the authors and co-cited authors analysis (Tables 6, 7), Inoue K and Tsuda M from Kyushu University were the top 2 most prolific authors with 61 and 60 articles respectively, Watkins LR from the University of Colorado System contributed 53 relevant studies, and Ji RR and Mika J with 50 and 48 articles ranked 4th and 5th, respectively. Among the co-cited authors, the top 5 authors, except for Tsuda M from Kyushu University, all work in different institutions from the US, indicating that the US possesses the leading researchers in microglia-related neuropathic pain. Tsuda M is the highest co-cited scholar, and their team focuses on the cellular and molecular mechanisms of microglia in neuropathic pain. Tsuda M’s team found that activation of P2X4Rs in spinal microglia triggered the release of BDNF from microglia and that pretreatment with interfering RNAs targeting BDNF could block the microglia–neuron signaling pathway to produce therapeutic effects (Coull et al., 2005). In addition, their team found that phosphorylation levels of p38 mitogen-activated protein kinase (p38MAPK) increased in ipsilateral dorsal horn microglia after nerve injury, revealing that activation of p38MAPK in spinal microglia is necessary for the development of tactile allodynia after nerve injury (Tsuda et al., 2004). Ji RR’s group has also made outstanding contributions in this area. They focused on the critical role of mitogen-activated protein kinases (MAPKs) in microglia signaling under neuropathic pain conditions. Ji RR’s team found in an earlier study that early activation of p38 in microglia in the ipsilateral dorsal horn of the spinal cord was induced by spinal nerve ligation (SNL) in adult rats, which facilitated the development of neuropathic pain (Jin et al., 2003). With outstanding contributions from Tsuda M’s and Ji RR’s teams, their study has the important significance of establishing a causal role for spinal microglia in neuropathic pain.

In the end, our visualization shows (Figure 4B) a low level of collaboration among core authors (or core teams) in microglia-related neuropathic pain studies. Collaborative research is a driving force for scientific progress and is an essential trend today. With continued research in this area, there is an opportunity for further collaboration and discussion between the different institutions and between the authors.

### The general structure of knowledge in microglia-related neuropathic pain

VOSviewer was used to examine the top 10 co-cited references and to construct a visual map. Six of the top 10 co-cited articles were basic studies, while the remaining four were review papers (Table 8). In particular, the article titled “Quantitative assessment of tactile allodynia in the rat paw” (495 citations) was the most cited (Chaplan et al., 1994). We found that almost all of the 10 highest co-cited articles were associated with microglia activation. Three of these studies revealed that microglia is key in initiating neuropathic pain (Jin et al., 2003; Raghavendra et al., 2003; Ledeboer et al., 2005). Indeed, among the interactions between neurons and glial, astrocyte-mediated neuroinflammation is a key mechanism for maintaining chronic pain such as neuropathic pain, whereas microglia activation may act in the earliest stages of neuropathic pain (Ji et al., 2013). The most co-cited article found that blocking spinal P2X4 receptors (P2X4Rs) by intrathecal injection of an antagonist reversed tactile allodynia caused by peripheral nerve injury (Tsuda et al., 2003). Notably, a review published by Milligan (Milligan and Watkins, 2009) in 2009 systematically summarized the simultaneous pathological and protective role of the glial in chronic pain. The authors shift their focus from reducing the glial activation to increasing the protective role of the glial, and discussing how the protective and anti-inflammatory effects of glial can be used in the future to develop new pharmacological targets to reduce neuropathic pain (Milligan and Watkins, 2009).

Combining Table 8 with the density map (Supplementary Figure S1), the highly co-cited 80 articles obtained were divided into 3 phases. In the initial phase (1980 to 2003) the neuroimmune action of microglia in neuropathic pain was the primary focus of research, and in the intermediate phase (2004 to 2009) neuron–glia interactions became a key mechanism for understanding the development and maintenance of neuropathic pain. According to current research (2010 to 2018), effectively addressing neuroinflammation may be critical to alleviating neuropathic pain. Furthermore, immune-targeted therapies have shown promising early evidence of success in treating neuropathic pain (Hutchinson et al., 2013). Not surprisingly, research into the mechanisms of microglia in neuropathic pain has gradually moved from basic research to the clinic.

### Identification of research hotspots and emerging topics

Visualization by a cluster of keywords analysis, time overlay maps, and density views (Figure 7). It can be seen that “activation of glial cells,” “MAP kinase,” “proinflammatory cytokine,” “central sensitization of pain,” “nociceptive hypersensitivity” and “neuroinflammation” are the main research directions for microglia in neuropathic pain.

Pain is considered to be an immune disorder. Microglia interact with cells such as neurons as immune cells of the central nervous system (Austin and Moalem-Taylor, 2010). Stimulation of increased levels of the pro-inflammatory cytokine IFN-γ (IFN-γR) after nerve injury resulted in the conversion of resting microglia into activated cells (Figure 6) and the generation of tactile allodynia. In particular, the authors suggest that decreasing IFN-γR-mediated signaling in spinal microglia may be a potential treatment for neuropathic pain (Tsuda et al., 2009). In addition, many studies have shown that chemokines play a key role in neuron–microglia communication in neuropathic pain. Among them, the most attention has been paid to the key signaling pair of fractalkine (FKN) and its receptor CX3CR1 (Verge et al., 2004; Clark et al., 2009; Yang et al., 2012). Thus, for physiological/pathological processes in the CNS, allowing the FKN/CX3CR1 signaling pair to be ideally located for regulating neuron–microglia communication. Studies have shown that chronic pain in rodents can be delayed or alleviated by intrathecal administration of neutralizing antibodies against FKN or CX3CR1 in different types of animal models of peripheral nerve injury (Milligan et al., 2004; Clark et al., 2007; Zhuang et al., 2007). And the downstream mechanism of FKN/CX3CR1 signaling may be mediated by microglia p38MAPK. By inhibiting CX3CR1, phosphorylation of p38MAPK in microglia is reduced, and ultimately the release of pro-inflammatory cytokines is reduced (Zhuang et al., 2007). In addition, deficits in neuropathic pain were observed in CX3CR1 knockout mice undergoing partial sciatic nerve ligation (PSNL) (Staniland et al., 2010). The evidence above suggests a specific strategy to inhibit microglia activation may hold considerable promise for the therapeutic administration of neuropathic pain. However, a more complex interaction exists between microglia and neurons.

It is known that macrophages can polarize into M1 cells with pro-inflammatory effects or M2 cells with anti-inflammatory functions (Wang et al., 2014). Similar to macrophage polarization, the polarization process of microglia is divided into an M1 phenotype, which expresses pro-inflammatory cytokines, or an M2 phenotype (David and Kroner, 2011), whose primary function is to alleviate inflammation and tissue repair (Orihuela et al., 2016). Therefore, the desirable way is to exert the anti-inflammatory and tissue repair capacity of microglia themselves by reducing the existence of the M1 microglia and/or converting them to the M2. Recent studies have demonstrated that some natural compounds such as parthenolide (Popiolek-Barczyk et al., 2015), dehydrocorydaline (Huo et al., 2018), and naringenin (Ge et al., 2022) could inhibit microglia-mediated neuroinflammation by stimulating the polarization of microglia to the M2, thereby attenuating allodynia and neuropathic pain in rats with bone cancer pain and rats with neuropathic pain. In addition, the endocannabinoid system and its ligand, 2-arachidonoylglycerol (2-AG), appear to be involved in the M1/M2 phenotypic switch in microglia during neuropathic pain. It was shown that the expression of CB2 receptors in microglia exhibited a consistency with the activation of microglia (Zhang et al., 2003; Racz et al., 2008; Guasti et al., 2009; Luongo et al., 2010). In addition, activation of CB2 receptors reduces nociceptive signal production and transmission by inducing a switch from the M1 phenotype to the M2 phenotype (Landry et al., 2012; Ma et al., 2015; Wu et al., 2019; Soliman et al., 2021). And 2-AG expression is significantly upregulated when microglia are converted to the M2 phenotype under pathological conditions (Mitrirattanakul et al., 2006; Mecha et al., 2015). A study elegantly confirmed that the endocannabinoid system is essential for the transformation of the M2 phenotype of microglia. It showed that cannabinoid receptor antagonists block microglia M2 polarization and that M2 polarization is dampened in CB2 receptor knockout mice (Mecha et al., 2015).

However, as multi-omics and single-cell techniques are increasingly used, the classical M1/M2 phenotypic classification may not fully explain the broad transcriptional state of microglia (Bennett et al., 2016). This landmark study (Bennett et al., 2016) showed that activated microglia clearly exhibit distinct phenotypes, but cannot be accurately distinguished by these phenotypes regarding the production of pro-inflammatory or anti-inflammatory mediators. This is due to the more complex biological properties of microglia, including region-specific, sex-dependent, and disease-specific activation properties (Kwon, 2022). Furthermore, multiple intermediate phenotypes can result from microglia function, depending on the microenvironment, the region, and the stage of the disease (Chiu et al., 2013). The M1/M2 phenotypes of activated microglia are typically mixed with one another, rather than showing a segregated condition, as seen in the pro-inflammatory effect of the M1 phenotype or the anti-inflammatory effect of the M2 phenotype. Microglia should therefore be considered dynamic cells that are plastic and strongly dependent on the context. New imaging tools and reporters will be needed in future studies to track changes in microglia over time, throughout their lifetime, and with treatment under different neuropathological conditions (Tay et al., 2017; Jordão et al., 2019).

A similar terminological aspect also involves “neuroinflammation” in this article (Figures 7A, 8). The meaning of the term “neuroinflammation” has often been equated with microglia “activation.” However, in practice, the definition of “neuroinflammation” often varies considerably between authors (Graeber, 2010). In fact, inflammation related to the CNS is usually a highly elaborated local reaction. Therefore, “neuroinflammation” may not directly refer to microglia “activation” (Woodburn et al., 2021). Moreover, the functions represented by the numerous transcriptional states of glial (including microglia, astrocytes, and oligodendrocytes) in the CNS are still not fully understood (Cahoy et al., 2008; Medina et al., 2022). In addition, “neuroinflammation” often implies deleterious effects (Aramideh et al., 2021). However, this terminology wrongly ignores the active role played by microglia in physiological functions. As a result, when we see the term “neuroinflammation” in the literature, we should understand what the authors are trying to convey in context rather than conflating it with the “activation” of microglia.

Taking into account the changes in the annual number of publications, we can broadly divide the evolution of keywords into two stages: Before 2009, the keywords “proinflammatory cytokine” (Ledeboer et al., 2005), “release of cytokines,” “spinal glial” (Wei et al., 2008), and “substances P” indicated the initial stage of studying microglia in neuropathic pain. From 2009 to 2015, the keywords “astrocytes” (Ji et al., 2013), “activation of glial” (Mika et al., 2013), “p38 MAPK” (Trang et al., 2009), “minocycline” (Ledeboer et al., 2005), and “opioid receptors” (Vacca et al., 2013) are extended directions based on previous research. From 2015 to 2021, the keywords “neuroinflammation” (Ji et al., 2018), “sex-differences” (Sorge et al., 2015), “satellite glial” (Lim et al., 2017), “NLRP3” (Grace et al., 2016), “mesenchymal stem cells” (Yang et al., 2020) have emerged as new research hotspots. According to the timeline analysis, it is clear that with the breakthroughs in new technologies in the biological sciences, research in this field is gradually shifting from cellular and molecular mechanisms to the exploration of new therapeutic techniques.

Burst analysis of references also reflects the hotspots and frontiers in a research field (Figure 6). The highest citation burst article is the 2018 review by Inoue K and Tsuda M in *Nature reviews neuroscience* (Inoue and Tsuda, 2018) (42.64, 2019–2021) highlighting spinal microglia’s pivotal role in developing pain hypersensitivity after nerve injury. The longest burst duration of basic research came from a landmark study published by Sorge et al. (2015) (24.8, 2016–2021), which found different dependencies of the pain hypersensitivity response on microglia and T cells between male and female mice through multiple experiments. In female mice, mechanical pain hypersensitivity is not exclusively microglia-mediated but is most likely mediated by the adaptive immune system (T lymphocytes). In previous pain studies, males have been used directly to denote both sexes, but research based on epidemiological and laboratory evidence indicates that chronic pain occurs more often in females (Mogil, 2012). This differentiated mechanism from the conventional view may help us understand why women have a higher risk of chronic pain. More importantly, microglia, T-lymphocytes, and other immune cells have been implicated to varying degrees in many neurological diseases, such as Alzheimer’s disease, Parkinson’s disease, and amyotrophic lateral sclerosis. Consequently, by transferring the concept of sexual dimorphism to the study of other neurological disorders, new insights into treating these diseases will be provided.

### Possible breakthrough for the roles of microglia in neuropathic pain

Old ideas are often overturned by technological advances. The above-mentioned debate about the M1/M2 phenotype of microglia is a typical example of this (Paolicelli et al., 2022): monolithic dichotomies are now perceived as overly simplistic and uncritical. Genomic, proteomic, spatial transcriptomic, and single-cell technologies have allowed us to better understand microglia function. Most current research on transcriptional changes in microglial heterogeneity focuses on neurodegenerative diseases (Masuda et al., 2020). In mouse models of Alzheimer’s disease (AD), there have been several different states of context-dependent microglia, also known as disease associated microglia (DAM) (Keren-Shaul et al., 2017). In the context of AD, microglia respond to amyloid pathology by transforming into DAM, downregulating genes such as Cx3cr1, Tmem119 and P2ry12 and upregulating genes such as Trem2, Apoe, and Ctsb have been observed (Keren-Shaul et al., 2017; Krasemann et al., 2017; Sala Frigerio et al., 2019). Despite this, little research has been conducted on microglial heterogeneous transcriptional changes in chronic or neuropathic pain. While DAM occurs in AD, microglial transcriptomic data have little or no correlation with the genes involved in DAM in neuropathic pain conditions (Denk et al., 2016; Jeong et al., 2016; Krasemann et al., 2017). Sideris-Lampretsas and Malcangio (2021), however, presented a different viewpoint, suggesting that microglia located near the ends of damaged nerve fibers are better able to adapt to neuronal changes, while microglia in adjacent neurons maintain a balanced state. Taking this perspective, it is exciting that microglia features associated with neuropathic pain have been identified. After peripheral nerve injury, microglia proliferate in the medial dorsal horn of the spinal cord and upregulation of specific genes (e.g., Ctss, Cx3cr1, Itgam, and Bdnf) related to neuropathic pain mechanisms occurs (Clark et al., 2007; Staniland et al., 2010; Guan et al., 2016). Nevertheless, more research is needed to investigate the transcriptional features of neuropathic pain-associated microglia in relation to their function. A unique microglial transcriptional profile that tends to completely represent neuropathic pain-related conditions may be uncovered by introducing advanced technologies and refining microglial studies across age, gender, region, disease, and species in the future. In the context of neuropathic pain, microglia will likely be investigated more thoroughly to enable new clinical therapeutic targets to be identified.

### Possibility of clinical translation

Combined with our research and findings, we believe introducing new therapeutic methods will largely change the paradigm of microglia research in neuropathic pain. Current treatments for pain rarely treat the disorder’s etiology; therefore, treatments for neuropathic pain often focus on reducing clinical symptoms (Gilron et al., 2015). However, current pharmacological and non-pharmacological treatments can only provide long-lasting pain relief for a very narrow range of patients (Alles and Smith, 2018), and these treatments are often coupled with various adverse effects (Moore et al., 2015). As a result, new therapeutic options, such as stem-cell therapy, are increasingly attracting the attention of scientists.

In recent decades, stem cells have shown remarkable anti-inflammatory and tissue repair efficacy (Peng et al., 2021), and their therapeutic effects may be associated with paracrine actions (Baraniak and McDevitt, 2010). Mesenchymal stem cells (MSCs) have become the preferred cells for cell therapy due to their high proliferative capacity, ability to differentiate into various tissue types and paracrine secretion of many factors with immunomodulatory properties (Ayala-Cuellar et al., 2019). Siniscalco et al. (2011) pioneered using human mesenchymal stem cells (hMSCs) to treat mice after nerve injury and systemic treatment with hMSCs administered via the tail vein produced an anti-nociceptive effect against injury. Following this, it has been shown that BMSCs transplantation reduces neuropathic pain caused by spinal cord injury by reducing the levels of p-p38 mitogen-activated protein kinase and extracellular signal-regulated kinase (p-ERK1/2) in spinal cord microglia (Watanabe et al., 2015). It has also been found that interleukin 1β-pretreated BMSCs inhibit spinal microglia activation through CCL7 mediation, thereby reducing neuropathic pain (Li et al., 2017). In addition, several studies have shown that BMSCs regulate spinal microglia by paracrine mechanisms to produce analgesia. The specific mechanisms involved may include the downregulation of P2X4R (Teng et al., 2019), the activation of the TLR2/MyD88/NF-κB pathway (Yang et al., 2020) and M2 phenotype microglia polarization in spinal microglia (Zhong et al., 2020). In another study, however, repeated intrathecal injections of MSCs after partial sciatic nerve ligation (PSNL) in rats did not produce a significant analgesic effect nor inhibit microglia activation in the ipsilateral spinal cord (Schäfer et al., 2014). It can therefore be speculated that the reasons for the uncertain therapeutic efficiency of MSCs may be related to their administration route. However, the risks of stem cell therapy are not limited to the uncertainty of the therapeutic outcome, as MSCs have the potential to cause pulmonary thrombosis or endogenous tumor formation (Jeong et al., 2011; Hua et al., 2022). In contrast, MSCs-derived extracellular vehicles (EVs), including exosomes, have the advantage of being readily available and stored and under few ethical restrictions compared to MSCs (Moghadasi et al., 2021). In addition, EVs/exosomes combine the advantages of cellular and nanotechnology in drug delivery (Liu et al., 2021). MSCs-derived EVs/exosomes in the cell-free treatment of neuropathic pain largely involve the participation of MicroRNAs. Intrathecal injection of MSC-EVs carrying miR-99b-3p reduced microglia activation in the spinal cord’s dorsal horn and mechanical allodynia in chronic constriction injury (CCI) rats by stimulating autophagy (Gao et al., 2023). Li et al. (2022) showed that the BMSC-derived exosomal miR-150-5p attenuates mechanical allodynia by targeting NOTCH2 in microglial. In addition to the beneficial action of MicroRNAs, Huc-MSCs-derived exosomes may exert analgesic effects on neuropathic pain by inhibiting activation of the TLR2/MyD88/NF-κB signaling pathway in spinal microglia (Gao et al., 2023).

Even though relevant studies are at a very early stage of clinical application, thanks to advances in technology and the increasing experimental data combined with clinical evidence to support them, we believe that stem-cell therapy and MSC-derived EVs/exosomes hold promise as potential treatment options for the clinical management of neuropathic pain.

## Limitation

This study is based on bibliometric analyses, using NC, NP, and H-index as the main research indicators and through visual analysis to help readers quickly grasp the current status of research and research trends as well as academic frontiers in this research field. Although our research is the initial bibliometric study of microglia-related neuropathic pain, there are a few limitations, as follows: (1) The WoSCC database is the most frequently accessed database during the scientometric analysis, and our search was therefore limited to literature in the WoSCC database, so some articles not included in the WoSCC were artificially omitted. Such limitations are also present in other bibliometric studies (Li et al., 2022; Wei et al., 2022). (2) Readers identify the most core content by a limited number of keywords (Ji et al., 2003; Basbaum et al., 2009; Costigan et al., 2009; Freeman, 2009; Jensen et al., 2011; Finnerup et al., 2015; Colloca et al., 2017) in the literature, which may lead to absent content extraction. (3) Citation indicators are time-dependent, which means that some of the best articles published more recently may lead to exclusion because they are less frequently cited. Consequently, there is a delay in reflecting up-to-date research. Despite all these limitations, it does not change the hotspots and trends revealed in this study. In conclusion, our research has unearthed useful information that may help researchers efficiently understand the research topics, trends, and academic hotspots of microglia in neuropathic pain.

## Conclusion

This study provides a bibliometric analysis and visualization of the literature focusing on the role of microglia in neuropathic pain from 2000 to 2021. We found that this field began to attract scholarly attention in 2004, and relevant research has been increasing yearly, especially in the last 5 years. It can be seen that the number of publications will be consistently increasing in the future. China and the United States are the top contributors in this area. The University of London is the highest publisher. The most productive author is Inoue K. *Journal of Neuroscience* is the journal with the highest total citations and average citations per article. In addition, current studies are focused on microglia activation, MAP kinase, and pro-inflammatory cytokines. The signaling between microglia and neurons is a core element of research in this field. Sexual dimorphism, neuroinflammation, and stem-cell therapy are possible frontiers in this field. Breakthroughs in these future key subjects will offer considerable promise for the clinical treatment of neuropathic pain. Overall, this study provides insights into the trends and characteristics of microglia-related neuropathic pain from a macroscopic perspective and offers valuable reference information for subsequent in-depth studies by other researchers.

## Data availability statement

The raw data supporting the conclusions of this article will be made available by the authors, without undue reservation.

## Author contributions

ZS-B designed the study. ZS-B and ZG-H searched and downloaded the data. ZG-H and LT-R re-checked the data. ZS-B and GC-Y analyzed the data. ZS-B drafted the manuscript. SY-Q, NW, and ZH-H reviewed the manuscript. All authors contributed to the article and approved the submitted version.

## Funding

This work was supported by the National Natural Science Foundation of China, Natural Science Foundation of China (31960175), Natural Science Foundation of Gansu Province (18JR3RA331), Fund Project of the Second Hospital of Lanzhou University (CY2017-MS06), and Gansu Youth Science and Technology Fund (20JR10RA752).

## Conflict of interest

The authors declare that the research was conducted in the absence of any commercial or financial relationships that could be construed as a potential conflict of interest.

## Publisher’s note

All claims expressed in this article are solely those of the authors and do not necessarily represent those of their affiliated organizations, or those of the publisher, the editors and the reviewers. Any product that may be evaluated in this article, or claim that may be made by its manufacturer, is not guaranteed or endorsed by the publisher.
