# Supplementary figures and images for: Bibliometric and visual analysis of microglia-related neuropathic pain from 2000 to 2021
Source: Front Mol Neurosci. 2023 May 18;16:1142852. doi: 10.3389/fnmol.2023.1142852 (PMC10233022; doi:10.3389/fnmol.2023.1142852)

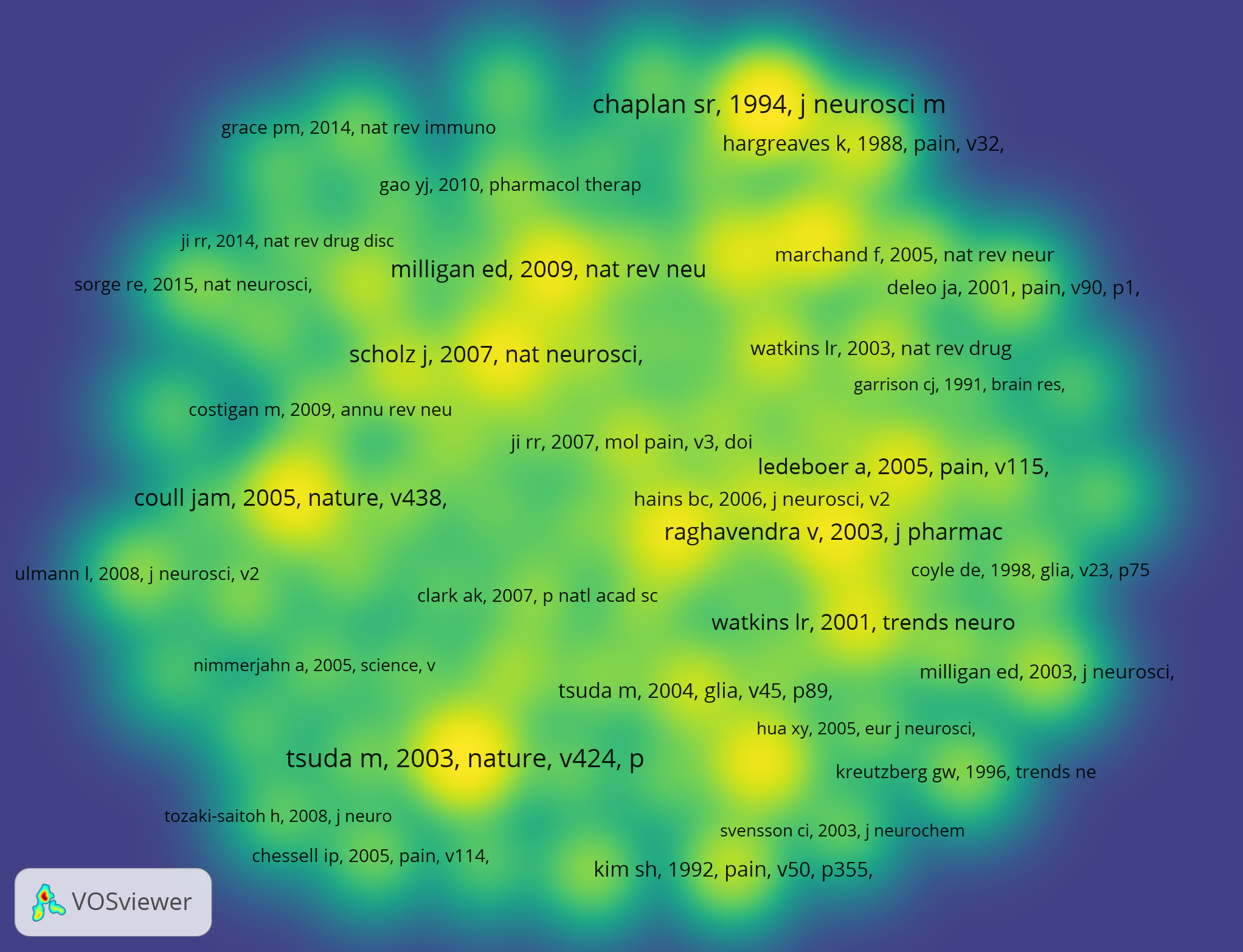

Supplement: Supplementary file 1 [file Image_1.TIFF]
